# Airway and parenchyma immune cells in influenza A(H1N1)pdm09 viral and non-viral diffuse alveolar damage

**DOI:** 10.1186/s12931-017-0630-x

**Published:** 2017-08-03

**Authors:** Monique Buttignol, Ruy Camargo Pires-Neto, Renata Calciolari Rossi e Silva, Marina Ballarin Albino, Marisa Dolhnikoff, Thais Mauad

**Affiliations:** Departament of Pathology, University of São Paulo - School of Medicine (FMUSP), Av. Dr. Arnaldo, 455 – 1 andar, sala 1155, São Paulo, SP 01246903 Brazil

**Keywords:** Diffuse alveolar damage, ARDS, Human Influenza, Pathology, Autopsy

## Abstract

**Background:**

Diffuse alveolar damage (DAD), which is the histological surrogate for acute respiratory distress syndrome (ARDS), has a multifactorial aetiology. Therefore it is possible that the immunopathology differs among the various presentations of DAD. The aim of this study is to compare lung immunopathology of viral (influenza A(H1N1)pdm09) to non-viral, extrapulmonary aetiologies in autopsy cases with DAD.

**Methods:**

The lung tissue of 44 patients, was divided in the H1N1 group (*n* = 15) characterized by severe pulmonary injury due to influenza A(H1N1)pdm09 infection; the ARDS group (*n* = 13), characterized by patients with DAD due to non-pulmonary causes; and the Control group (*n* = 16), consisting of patients with non-pulmonary causes of death. Immunohistochemistry and image analysis were used to quantify, in the parenchyma and small airways, several immune cell markers.

**Results:**

Both DAD groups had higher expression of neutrophils and macrophages in parenchyma and small airways. However, there was a higher expression of CD4+ and CD8+ T lymphocytes, CD83+ dendritic cells, granzyme A+ and natural killer + cell density in the lung parenchyma of the H1N1 group (*p* < 0.05). In the small airways, there was a lower cell density of tryptase + mast cells and dendritic + cells and an increase of IL-17 in both DAD groups (*p* < 0.05).

**Conclusion:**

DAD due to viral A(H1N1)pdm09 is associated with a cytotoxic inflammatory phenotype, with partially divergent responses in the parenchyma relative to the small airways. In non-viral DAD, main immune cell alterations were found at the small airway level, reinforcing the role of the small airways in the pathogenesis of the exudative phase of DAD.

**Electronic supplementary material:**

The online version of this article (doi:10.1186/s12931-017-0630-x) contains supplementary material, which is available to authorized users.

## Background

Diffuse alveolar damage (DAD) is a histological pattern characterized by hyaline membranes, intra-alveolar oedema, alveolar epithelial cell injury, and neutrophilic inflammation. DAD is commonly found in patients with acute respiratory distress syndrome (ARDS), and it is considered a pathological hallmark that is correlated with the severity and duration of the disease [1–3].

DAD can occur as a consequence of different forms of lung insult, such as bacterial and viral infections, connective tissue diseases and sepsis, among others. In addition, the aetiology of DAD has been broadly divided in two groups: lung injury that affects primarily the pulmonary parenchyma (pneumonia, aspiration of gastric contents, inhalation injury); or indirect lung injury in the setting of a systemic process with an acute systemic inflammatory response (sepsis, severe trauma, pancreatitis) [4, 5].

Neutrophils and macrophages have been recognized as the main drivers in the physiopathology of DAD [6], resulting in the release of several cytokines associated with direct injury to the alveolar-capillary membranes. However, other cells from the innate and adaptive immune systems, such as lymphocytes, mast cells and dendritic cells, also certainly play roles in the pathogenesis of DAD, as shown mainly in animal studies [7, 8]. Due to its multiple aetiologies, it is plausible to conceive that the immunopathology is different in the various presentations of DAD.

The pandemic influenza A(H1N1)pdm09 virus emerged in 2009 and spread globally, leading to 284,400 deaths in that year [9]. Since then, the virus has circulated worldwide with an *skip-and-resurgence* behaviour in recent years [10]. A new outbreak of cases was observed in May 2016 in Brazil, mainly in São Paulo, with 411 confirmed deaths. [11]. Severe infection caused by the influenza A(H1N1)pdm09 virus can induce ARDS with a histopathological pattern of DAD associated with bronchiolitis and haemorrhage, as previously shown by our group [12]. The clinical course of influenza A(H1N1)pdm09-associated ARDS is different from non-H1N1 ARDS, in which patients require more extensive therapy, including extracorporeal lung support [13].

To date, very few pathological papers have analysed the broad panel of cells related to innate and adaptive immunity in the lungs of critical patients with DAD. We hypothesized that a viral cause of DAD would be associated with the presence of cytotoxicity-associated cells, which would be not present in the non-viral, non-pulmonary causes of DAD. Since airways and alveoli present site-specific differences in leukocyte trafficking and regulation of inflammations [14], we further reasoned that small airways would present a different profile than that of the parenchyma. Therefore, the aim of this study was to describe, quantify and compare the immune cells in the alveolar parenchyma and small airways of patients with viral and non-viral causes of DAD.

Some of the results of this study have been previously reported in the form of abstracts [15–18].

## Methods

This study was approved by the institutional review board of São Paulo University Medical School. It was a retrospective study using archived material from routine autopsies performed by the Autopsy Service of the Department of Pathology of Sao Paulo University Medical School.

### Study population

Lung tissue from 44 autopsies performed at Sao Paulo University Medical School between 2002 and 2010 was retrospectively included in this study and was divided into three groups according to the cause of death. For all cases, autopsy reports and clinical charts were reviewed. We only included cases with clinical diagnosis of ARDS (ARDS group) or histological diagnoses of diffuse alveolar damage (H1N1 group) or absence of any pulmonary disease (control group), and with sufficient archived lung tissue material (at least 20 mm of alveolar septum).

The H1N1 group (*n* = 15) was characterized by pulmonary injury due to influenza A(H1N1)pdm09 infection. Viral infection was confirmed by nasopharyngeal swab specimens or in lung tissue using real-time reverse transcriptase polymerase chain reaction (rRT-PCR) testing, in accordance with guidelines from the Centers for Disease Control and Prevention (CDC).

The ARDS group (*n* = 13) was characterized by non-pulmonary ARDS patients with non-viral infection causes of death, with histological findings of DAD [19] and the absence of chronic lung diseases. Clinical diagnosis of ARDS was defined accordingly to the Berlin definition [20].

The Control group (*n* = 16) was characterized by a non-pulmonary cause of death among non-smokers and non-ventilated patients and without previous lung diseases. All of these patients showed normal lung architecture on gross and microscopic examinations.

Lung tissue of these patients have already been used in previous studies of our group [12, 21, 22].

### Tissue processing

Paraffin blocks of lung tissue collected during routine autopsy were retrieved from the archives of the Department of Pathology of Sao Paulo University Medical School. Three to four fragments of lung tissue were randomly collected from regions of altered lung parenchyma. In normal lungs, one fragment of lung tissue was collected from each lobe. The tissue was previously fixed in 10% buffered formalin for 24 h, routinely processed and embedded in paraffin. Five-micrometre-thick sections were stained with haematoxylin and eosin (H&E) for confirmation of the histological diagnosis of DAD and the identification of small airways.

### Morphological analysis and immunohistochemistry

One to three slides per patient containing alveolar septa and small airways were selected for analysis and immunohistochemical staining.

Immunostaining for the following markers was performed: CD8 T cells, CD4 T cells, CD83 cells, CD207 Langerhans cells, CD 57 natural killer cells (NK cells), chymase and tryptase mast cells, FOXP3 regulatory T cells, IL-17, Neutrophil elastase, CD 68 macrophages, and granzyme A and B cells, as previously described [23]. Antibody types and the pre-treatments used are shown in Additional file 1: Table S1.

After immunohistochemical staining, each slide was scanned using Panoramic Viewer® software, version 1.15.2 for Windows® (3DHistech, Budapest, Hungary).

For the alveolar parenchyma analyses, approximately 20 fields per case were selected for image analysis, with each field containing 1000 μm of alveolar septum at 500× magnification. Small airways were defined as those showing a basement membrane (BM) perimeter ≤6 mm [24]. Two to four small airways were analysed per patient, and the density of the inflammatory cells was quantified in each airway. The entire circumference was analysed at 500× magnification.

Marker expression in small airways and parenchyma was assessed using Image-Pro Plus® image-analysis software, version 6.0 for Windows® (Media Cybernetics, Silver Spring, MD, USA).

The expression of the different markers in the alveolar septa was calculated as the density of positive cells divided by the corresponding alveolar septum length (cells/μm) and in the airways as the positive cells divided by the basal membrane perimeter (cells/μm). Since macrophages and neutrophils are present also in alveolar spaces, we quantified the density of these cells present in alveolar walls and in alveolar spaces, divided by the corresponding alveolar length. All of the slides were previously coded and analysed by two investigators blinded to the study groups. The interobserver correlation was kappa = 0.81.

### Statistical analysis

Statistical analysis was performed using the SPSS statistical software package, version 15 for Windows (SPSS, Chicago, IL, USA), and GraphPad Prism software, version 5.00 for Windows (GraphPad Software, San Diego, CA, USA). Data are presented as the means ± SDs or medians (interquartile ranges). After testing for the distribution of data, *one-way ANOVA* or *Kruskal Wallis tests* were used to compare data among the H1N1, ARDS and Control groups, with adjustments using Bonferroni’s test. The association between cell densities was analysed by Pearson’s or Spearman’s coefficient tests. The level of significance was set at *p* ≤ 0.05.

## Results

### Study population

Demographic and clinical data of the H1N1 (*n* = 15), ARDS (*n* = 13) and Control (*n* = 16) group are presented in Table 1. The mean ± SD age of the H1N1, ARDS and Control group patients were 50 ± 12, 43 ± 15, and 54 ± 15 years old, respectively (*p* = 0.133). The main cause of death in the ARDS group was refractory sepsis (46%), and the primary causes of death in the Control group were cardiovascular diseases (56%).

**Table 1:** Demographic and clinical data

Characteristics	Control (*n* = 16)	ARDS (*n* = 13)	H1N1 (*n* = 15)
Age (years, mean ± SD)	54 ± 15	43 ± 15	50 ± 12
Sex (male/female)	9/7	5/8	9/6
Primary cause of death, n (%)			
Respiratory failure	-	4 (30)	11(73)
Cardiovascular diseases	9 (56)	-	2 (13)
Liver diseases	4 (25)	-	-
Refractory sepsis	-	6 (46)	2 (13)
Gastrointestinal disease	2 (12)	-	-
Gastrointestinal bleeding	1 (6)	3 (23)	-

Patients with non-pulmonary ARDS showed the classical histological picture of DAD, characterized by hyaline membranes, intra-alveolar oedema, and neutrophilic exudates. The histological pattern observed in all of the patients was the exudative pattern, with four of them showing an initial proliferative pattern. There was no extensive tissue proliferation or fibrosis. All of the patients were ventilated using a lung-protective strategy with a low tidal volume (≤6 mL/Kg); the ventilatory parameters and PaO_2_/FiO_2_ values of ARDS are presented in Table 2. Three patients presented secondary bronchopneumonia according to the autopsy reports.

**Table 2:** Days of evolution, ventilatory parameters, PaO2/FiO2 values and predisposing factors for acute respiratory distress syndrome (ARDS) patients

Patient	Days of ARDS	Driving pressure cm H_2_O	PEEP cm H_2_O	PaO_2_/FiO_2_	Predisposing factors
1	1	10	8	161	Acute fulminant hepatitis
2	1	6	6	170.7	Cirrhosis
3	1	12	9	93.7	Lupus erythaematosus with hypovolaemic shock
4	2	25	10	166.3	Chronic pancreatitis with cholangitis
5	2	15	8	130.8	Cirrhosis; diarrhoea
6	2	13	14	156.5	Disseminated lymphoma
7	2	10	11	111.7	Myelitis
8	3	20	18	84	Cirrhosis
9	4	14	11	197	Multiple myeloma, acute gastrointestinal bleeding
10	6	15	10	196.7	Acute mesenteric ischaemia
11	9	12	17	119.7	Infectious endocarditis
12	10	16	14	108.3	Cirrhosis with variceal bleeding
13	16	10	10	186.7	Inguinal abscesses with peritonitis
Mean ± SD	4.5 ± 4.5	13 ± 4.7	11 ± 6.2	144 ± 39	

*Days of ARDS* mean period from ARDS diagnosis until death. PEEP corresponds to mean values in the first 48 h, *PaO2/FiO2* ratio of arterial oxygen tension to the fraction of inspired oxygen, *PEEP* positive end-expiratory pressure. PaO2/FiO2 corresponds to values assessed at the time of clinical diagnosis for each patient

All of the patients with influenza A(H1N1)pdm09 infection showed extensive exudative DAD with variable degrees of pulmonary haemorrhage and necrotizing bronchiolitis. Six patients had evidence of bacterial infection in the lungs, confirmed by the presence of positive histochemical staining for bacteria, positive culture in the bronchial aspirate and/or positive PCR. Both PCR and bronchial aspirate cultures detected *Streptococcus pneumoniae* in all of the patients [12]. Patients with normal lung histology were used as controls.

**Fig. 1:**
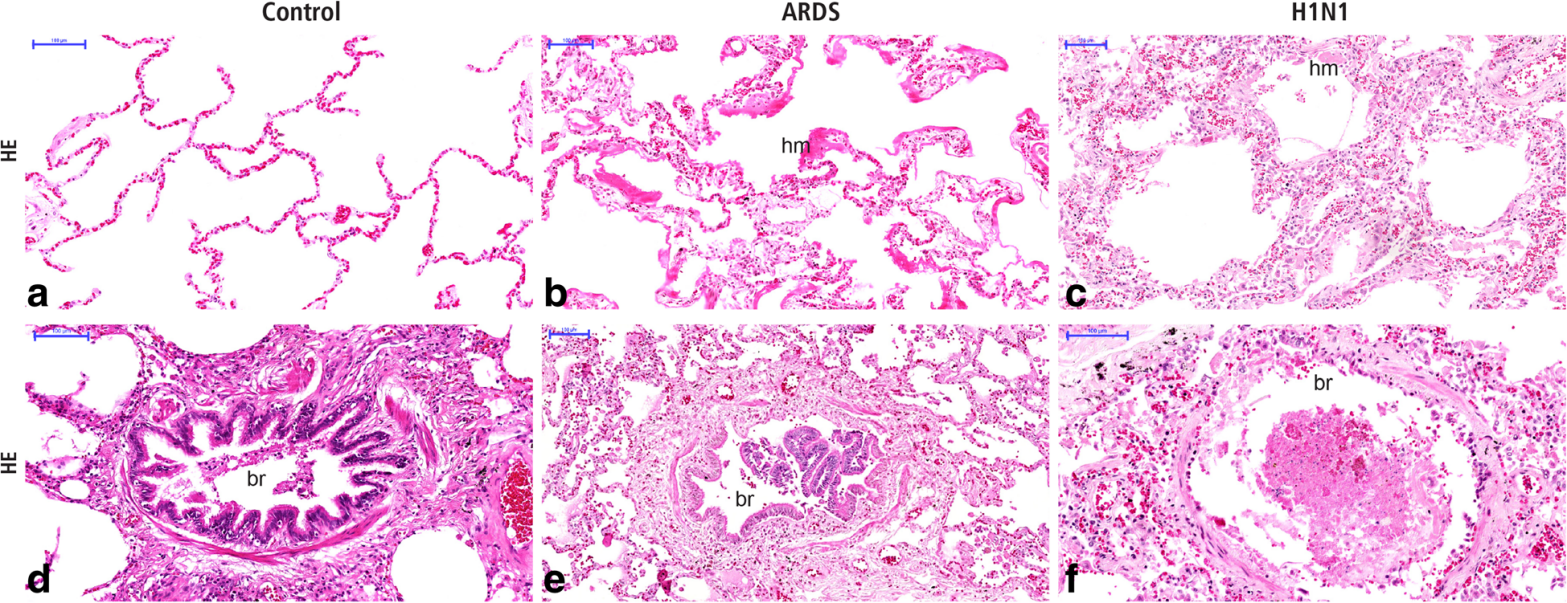
Representative H&E photomicrographs of the lung parenchyma from the Control **a** ARDS **b** and H1N1 **c** groups. Observe the presence of hyaline membranes (hm) in the ARDS group and inflamed and oedematous alveolar septa with hyaline membranes (hm) in the H1N1 group. Representative photomicrographs of the small airways from the Control **d**, ARDS **e** and H1N1 **f** groups, stained with H&E. There is small airway (br) oedema with epithelial denudation and the presence of inflammatory cells in the bronchioles (br) in the ARDS and H1N1 groups

Representative histological pictures of control and DAD groups are shown in Fig. 1.

### Morphological analysis

A mean of 26.4 ± 8.1 mm of alveolar septum was analysed by staining for each patient. There were no differences in total septum length among the H1N1, ARDS and Control groups (25.7 ± 7.4 mm, 28.3 ± 5.2 mm, 25.3 ± 10.6 mm, respectively, *p* = 0.583).

Because of extensive necrotizing bronchiolitis in the H1N1 patients and extensive tissue use for the other groups, not all of the cases had the same number of small airways analysed, with some cases having no airways suitable for analysis. A total of 1161 small airways were analysed considering all of the stainings used, the number of analyzed cases for each marker varied from 6 to 13 in Control, 10–13 in ARDS and 6–13 in H1N1 cases. The mean perimeter of the small airways for the H1N1, ARDS and Control groups were 2.4 ± 0.6 mm, 2.3 ± 0.7 mm, 2.1 ± 0.9 mm, respectively, corresponding to small membranous bronchioles (*p* = 0.70). Representative images of the positive stained cells are shown in the Additional file 2: Figure S1.

**Fig. 2:**
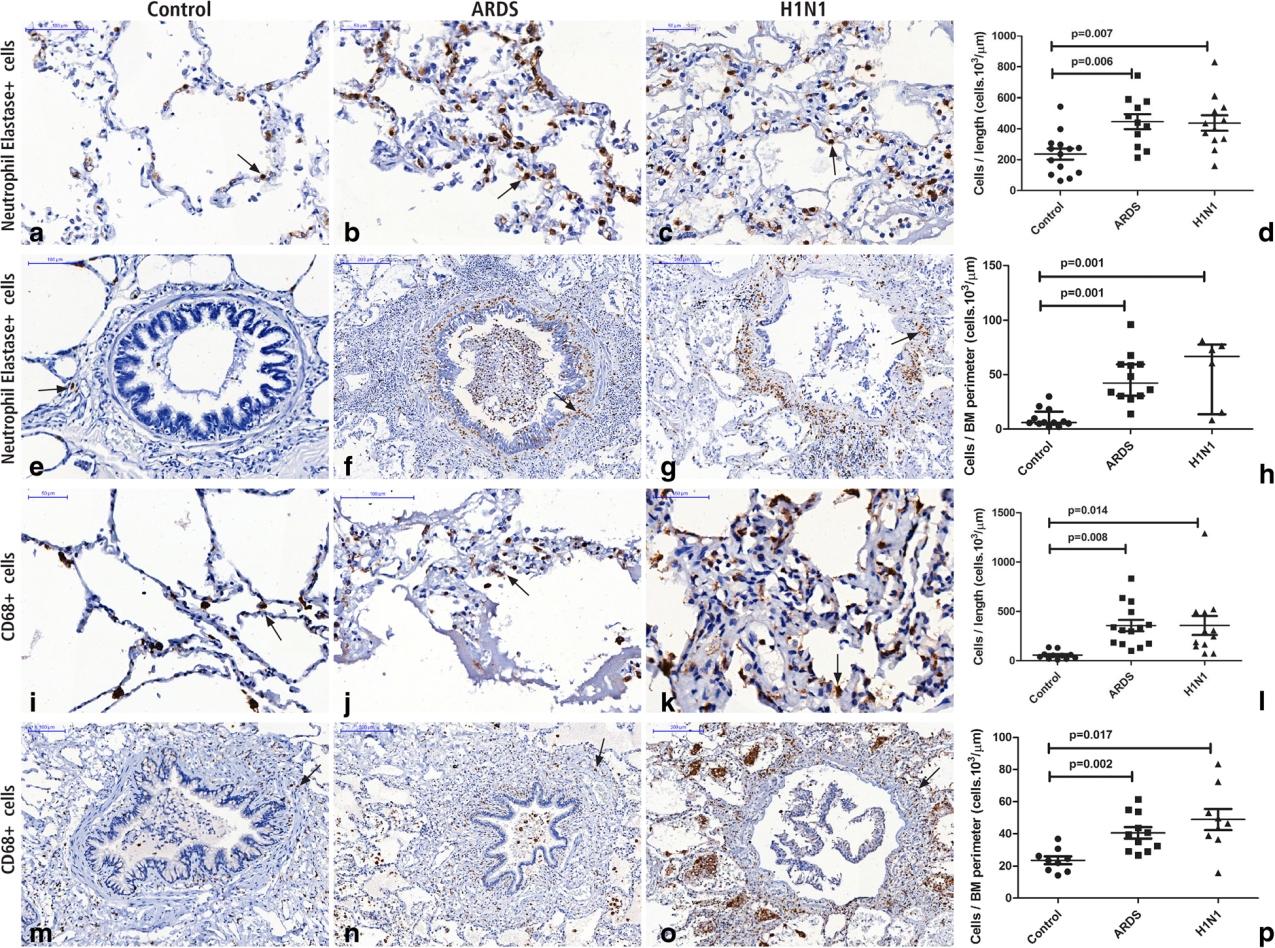
Representative photomicrographs from the Control, ARDS and H1N1 groups showing Neutrophil Elastase + cells in lung parenchyma (**a**, **b**, **c**), and small airway (**e**, **f**, **g**); and CD68+ macrophages in lung parenchyma (**i**, **j**, **k**), and small airways (**m**, **n**, **o**). There was increased expression of Neutrophil Elastase+ cells and CD68+ macrophages in the H1N1 and ARDS groups compared to the Control group, in the parenchyma and small airways. The arrows indicate positive stainings. Graphs show the density of Elastase+ cells (**d**, **h**) and CD68+ macrophages (**l**, **p**) in the Control, ARDS and H1N1 groups (one-way ANOVA and Kruskal Wallis test)

Given the major role of neutrophils and macrophages in the pathophysiology of DAD, these cells were quantified in lung parenchyma and small airways, with a significantly higher density of cells in alveolar septa and alveolar spaces in ARDS and H1N1 when compared to the controls as expected. There were no differences between DAD groups (Fig. 2).

**Fig. 3:**
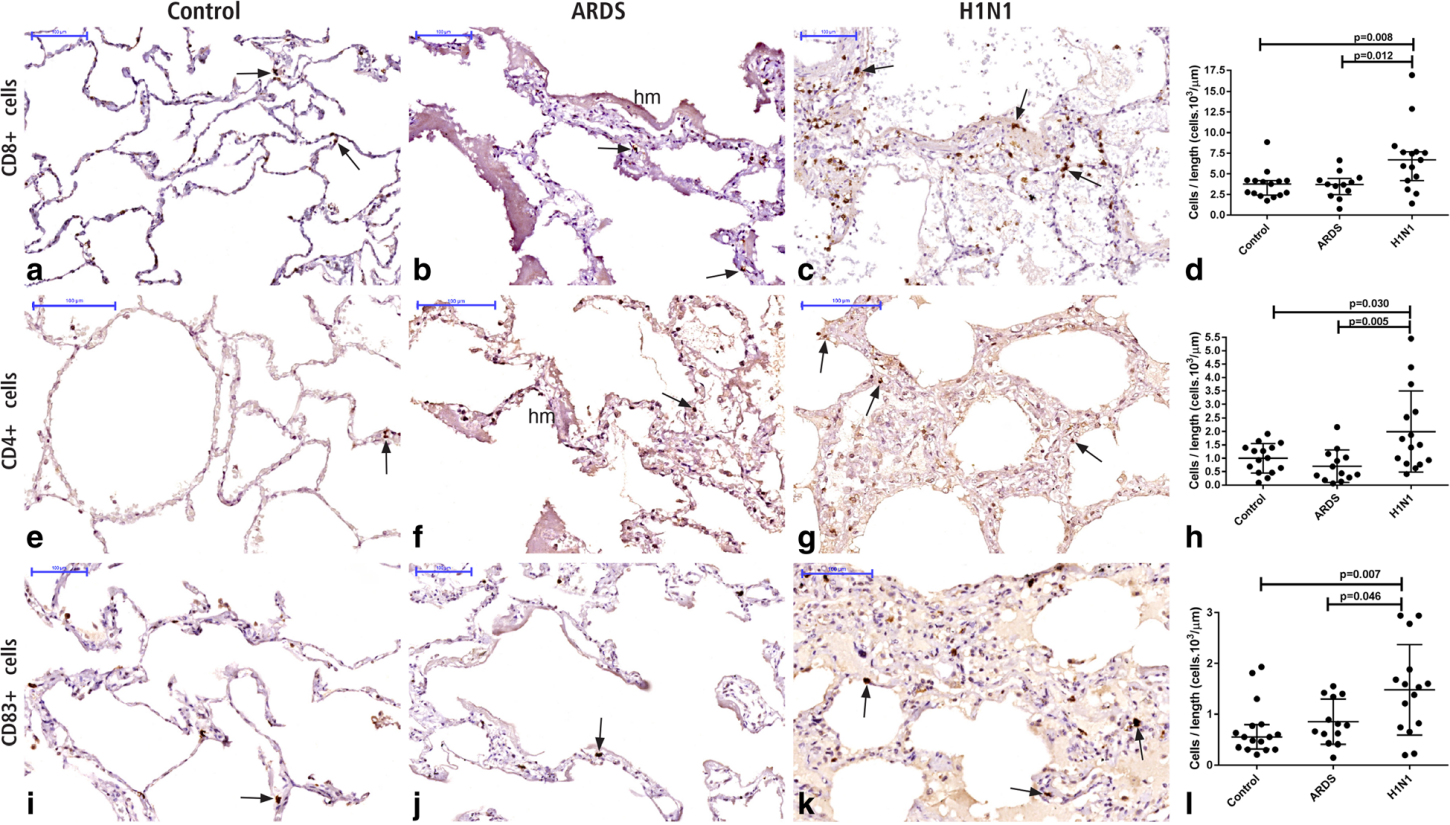
Representative photomicrographs of the lung parenchyma from Control, ARDS and H1N1 groups, stained immunohistochemically with CD8+ T cells (**a**, **b**, **c**), CD4+ T cells (**e**, **f**, **g**), CD83+ (**i**, **j**, **k**). There was increased expression of CD8+ T cells, CD4+ T cells and CD83+ cells in the H1N1 group compared to the ARDS and Control groups. The arrows indicate positive stainings. Scale bar = 100 μm. Graphs show the density of, CD8+ T cells (**d**), CD4+ T cells (**h**), CD83+ dendritic cells (**l**) of the Control, ARDS and H1N1 groups (*one-way ANOVA* and *Kruskal Wallis* test)

**Fig. 4:**
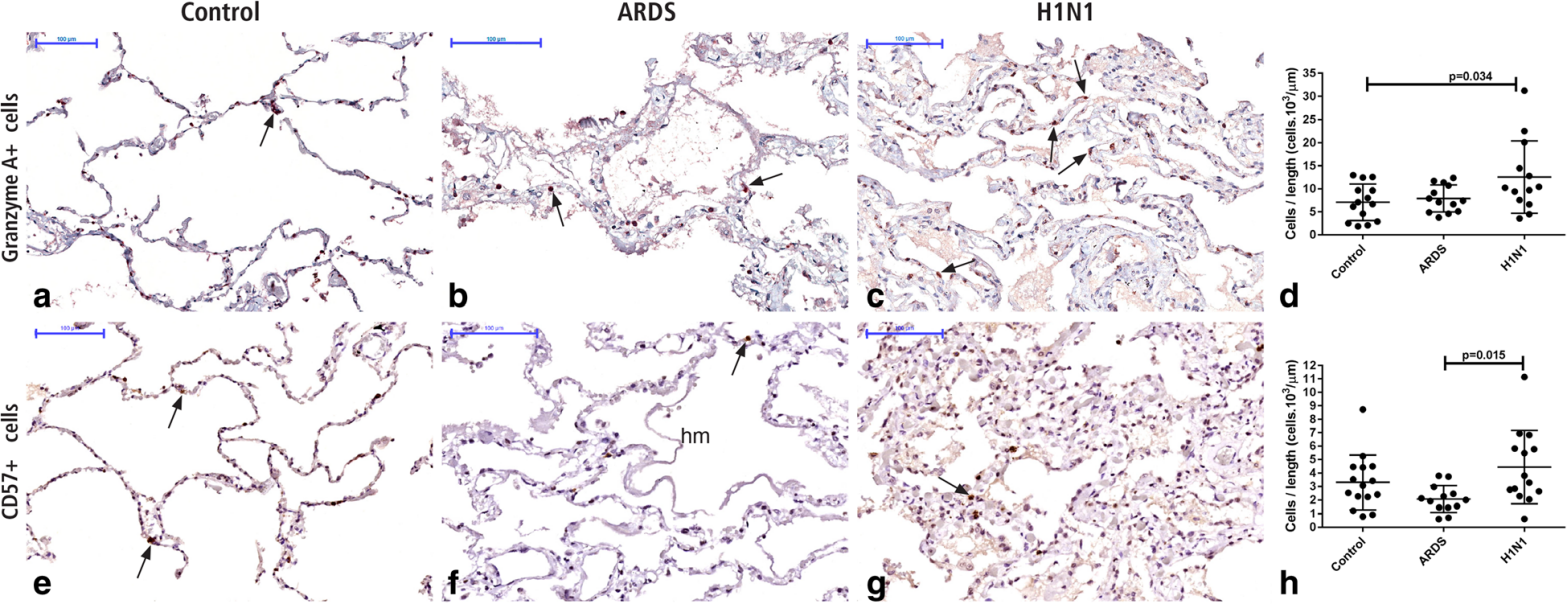
Representative photomicrographs of the lung parenchyma from the Control, ARDS and H1N1 groups, stained immunohistochemically granzyme A (**a**, **b**, **c**) and CD57 (**e**, **f**, **g**) antibodies. There was increased expression of granzyme A+ cells in the H1N1 group, compared to the Control group; and increased expression of CD57+ cells in the H1N1 group, compared to the ARDS group. The arrows indicate positive staining. Scale bar = 100 μm. Graphs show the density of granzyme A+ cells (**d**) and CD57+ cells (**h**) in the parenchyma of the Control, ARDS and H1N1 groups (*one-way ANOVA* and *Kruskal Wallis* test)

In lung parenchyma analysis, there was a significantly higher expression of CD8+ T cells (Fig. 3 a, b, c, d), CD4+ T cells (Fig. 3 e, f, g, h), and CD83+ cells (Fig. 3 h, i, j, k) in the H1N1 group compared to the ARDS and Control groups. In addition, there was higher expression of granzyme A+ cell (Fig. 4 a, b, c, d) density in the H1N1 group, compared to the Control group. CD57+ (Fig. 4 e, f, g, f) cell density was increased in the H1N1 group, compared to the ARDS group.

There were no significant differences among the groups in the expression of chymase + and tryptase + mast cells, granzyme B+, IL-17 or CD20+ B cells, shown in Additional file 1: Table S2.

**Fig. 5:**
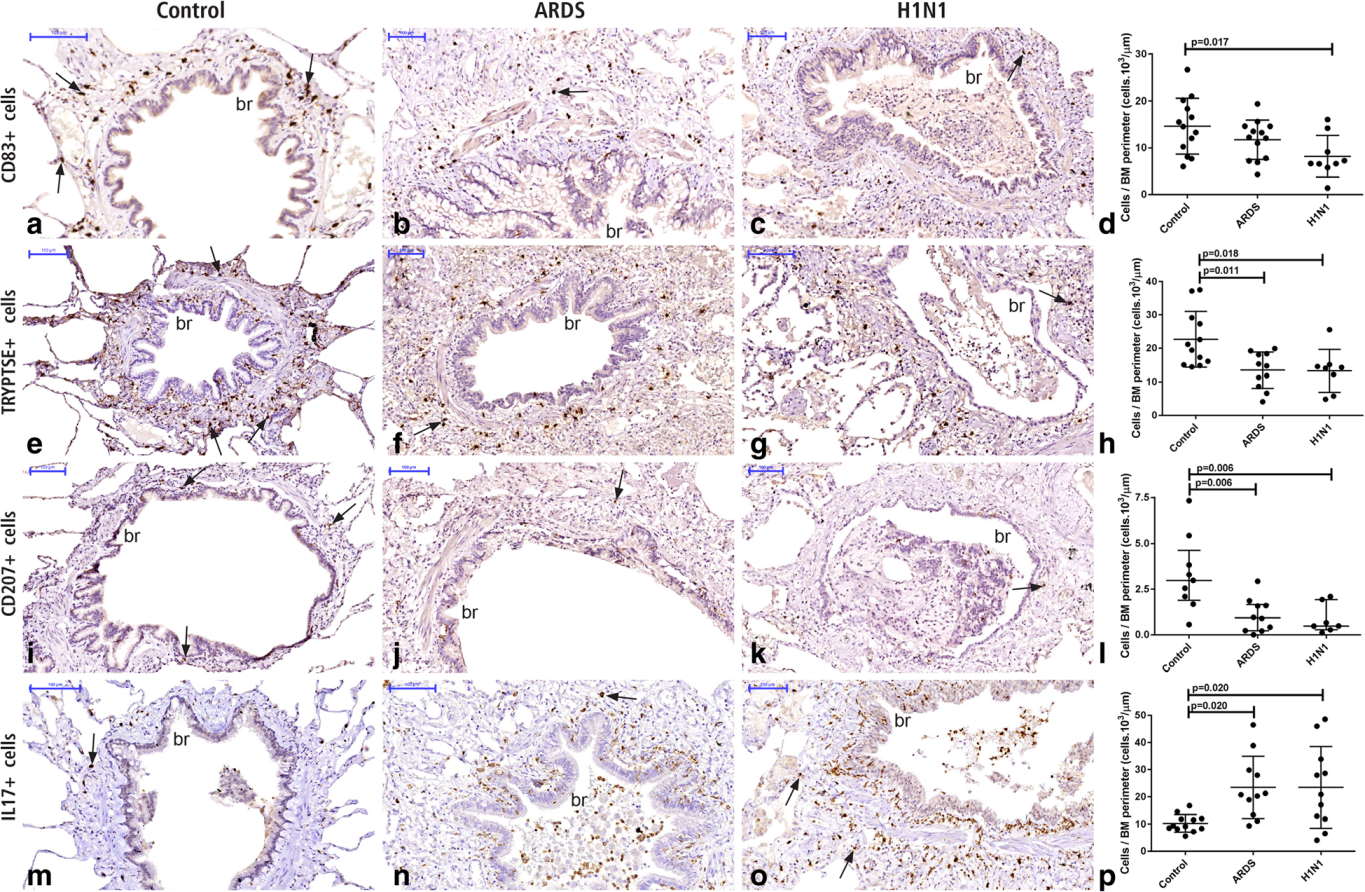
Representative photomicrographs of the small airways from the Control, ARDS and H1N1 groups, stained immunohistochemically with CD83 (**a**, **b**, **c**), mast cell tryptase (**e**, **f**, **g**), CD207 (**i**, **j**, **k**), and IL-17 (**m**, **n**, **o**) antibodies. There was decreased expression of CD83+ cells in the H1N1 group compared to the controls, decreased expression of tryptase + mast cells and CD207+ cells in the H1N1 and ARDS groups, compared to the controls, and increased expression of IL-17 in the H1N1 and ARDS groups compared to the controls (arrows). The arrows indicate positive stainings. Scale bar = 100 μm. Graphs show the density of CD83+ dendritic cells (**d**), tryptase + mast cells (**h**), CD207+ Langerhans cells (**l**), and IL-17+ cells (**p**) in the small airways of the H1N1, ARDS and Control groups (*one-way ANOVA* and *Kruskal Wallis* test)

In small airway analysis, there was significantly decreased expression of CD83+ cells (Fig. 5 a, b, c, d) in the H1N1 group, compared to the controls. There was decreased expression of tryptase + mast cells (Fig. 5 e, f, g, h) and CD207+ cells (Fig. 5 i, j, k, l) in the H1N1 and ARDS groups, compared to the controls. Conversely, IL-17+ (Fig. 5 m, m, o, p) was increased in the H1N1 and ARDS groups compared to the Control group.

There were no significant differences among the groups in the expression of CD8+ T cells, CD4+ T cells, CD57+ NK cells, chymase + mast cells, granzyme A and B or CD20+ B cells, shown in Additional file 1: Table S3.

The density of positive cells for Foxp3+ was very low in all of the samples and therefore was not quantified; the same finding occurred for CD207+ cells relative to the alveolar parenchyma.

There were no differences between patients with the exudative and initial proliferative histological patterns when the cellular analysis and clinical and ventilatory parameters in the ARDS group (*p* > 0.05) were compared, data not shown. In addition, no statistical differences were found when cases with bronchopneumonia in both H1N1 and ARDS groups were compared with cases without bronchopneumonia (*p* > 0.05), data not shown.

### Morphological correlations

**Fig. 6:**
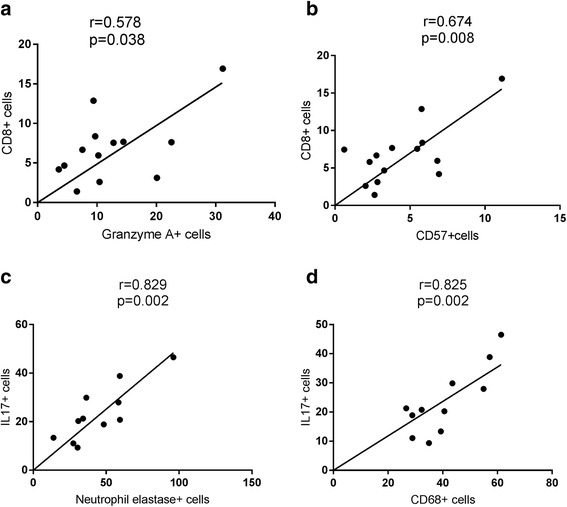
Graphs show the positive correlations between the cell density of granzyme A+ and CD8+ cells (**a**), CD57+ and CD8+ cells (**b**) in the lung parenchyma of H1N1 patients; and neutrophil elastase + and IL17+ cells (**c**), and CD68+ cells (**d**) and IL17+ cells in the small airways of ARDS patients (Pearson’s coefficient tests)

There was a positive correlation of the cell density of CD8+ cells with granzyme A+ cells (*r* = 0.578; *p* = 0.038) and CD57+ cells (*r* = 0.674; *p* = 0.008) in the parenchymas of H1N1 patients. There were a significant correlation between IL17+ cells and neutrophils (*r* = 0.829, *p* = 0.002) and macrophages (*r* = 0.825, *p* = 0.002) in the small airways in the ARDS group. The correlations are shown in Fig. 6. There were no significant correlations between immune cells and clinical/ventilatory parameters in ARDS group.

## Discussion

In the present study, we analysed a broad panel of cells related to innate and adaptive immunity in the lungs of critical patients with DAD. Our main finding was that DAD due to influenza A(H1N1)pdm09 virus infection was associated with an inflammatory phenotype different from non-viral causes of DAD, with partially divergent responses in the parenchyma relative to the airways. Both DAD groups presented higher alveolar and airway densities of neutrophils and macrophages. However, there was a significantly higher expression of CD4+ and CD8+ T lymphocytes, dendritic cells, granzyme A+, and NK cell density in the parenchyma of patients who died from influenza A(H1N1)pdm09 DAD, and there was lower cell density of tryptase + mast cells dendritic + cells and an increased number of IL-17+ cells in the airways, compared to non-viral DAD cases and the controls. These findings extend our previous description of the immunopathology on influenza A(H1N1)pdm09 severe lung infection, adding quantitative parameters to a broad cellular panel of immune cells, compared to other causes of DAD in different lung compartments.

In contrast, no significant changes in immune cells other than neutrophils and macrophages at the parenchymal level were observed in non-viral DAD cases compared to the controls. At small airway level there was a decreased cell density of tryptase + mast cells and CD207+ Langerhans cells and an increase in IL-17+ cells. Our data confirm that the effector cells neutrophils and macrophages are important in the pathogenesis of DAD [25]. The lack of other immune cell alterations at the parenchyma level in non-viral DAD causes could be explained by most of them having an extrapulmonary etiology with lymphocytic related immunological phenomena probably not occurring in the lungs. To our knowledge, this study was the first to compare immune cell infiltration in the parenchyma and airways of patients with DAD of different aetiologies.

As expected, immune responses in influenza A(H1N1)pdm09-associated DAD presented cytotoxic characteristics with increases in CD8+ T cells, NK+ cells and granzyme A+ cells in the parenchyma region, with strong correlations between CD8+ and CD57+ cells in the parenchyma. Granzymes are a family of five serine proteases present in the granules of cytotoxic cells, which require perforin to exert their cytotoxic activities, inducing apoptosis by different pathways. Granzyme A+ is more abundant, but granzyme B+ has more potent cytotoxic activity [26]. We previously described the presence of granzyme B+ cells in the lungs of these patients [12], but the current quantitative analysis showed that granzyme A+ was actually increased in the parenchyma with strong correlations between granzyme A and CD8+ cells. Harari et al. previously described in humans the blood profiles of CD8 T cells specific to influenza viruses, showing that CD8+ T cells were mostly perforin^−^, granzyme K^+^, granzyme A^+/−^/, and granzyme B^−^ and that granzyme A was not associated with a cytotoxic profile [27]. Hirota et al. previously described the presence of increased granzyme B+ cells in the lungs of ARDS patients derived from biopsies and autopsies, but the causes of ARDS were not presented in their paper [7]. Whether the observed granzyme A profiles of influenza A(H1N1)pdm09 cases were associated with less potent cytotoxic capacity and, therefore, with increased disease severity deserves further investigation.

There was a divergent pattern in the density of dendritic cells in A(H1N1)pdm09-associated DAD in the parenchyma vs. the airways. We used CD83 as a marker of mature dendritic cells, but CD4(+)CD25(+) regulatory T Foxp3+ cells can also express CD83 [28]. Since in this study there were negligible numbers of Foxp3+ cells in the lungs, it is likely that most of the CD83 + cells were mature dendritc cells (DCs). Whereas an increase in CD83+ matureDCs were observed in the parenchyma, SA presented a decrease in CD83+ DCs relative to the controls and a decrease in CD207+ Langerhans DCs relative to the controls and to non-viral DAD. This pattern was previously described in an experimental model of influenza A infection in mice, in which depletion was observed in CD11b + and CD11b2 myeloid DC subsets in the airway mucosa at day 4 of infection, returning to baseline by day 14 post-infection [29]. In the parenchyma, DCs were significantly elevated at day 14 and remained so until day 21 post-infection. Because airway DCs tend to have first contact with aeroallergens, it has been speculated that this early decrease is due to enhanced migration to draining LNs or apoptosis. Parenchymal DCs in the alveolar walls would represent a distinct microenvironment, which would be important in the later phases of infection, regulating T cell immunity [30]. To our knowledge, this study was the first to show a similar pattern in human tissue.

We found decreased cell density of tryptase + mast cells in the small airways of both DAD groups. Mast cells are important cells in the surveillance of infection, and H1N1 virus can infect mast cells, stimulating the release of cytokines and chemokines [31]. In contrast, Liu et al. showed that H1N1 virus induced mast cell apoptosis, which could explain our findings [32]. In non-viral DAD, the reasons for the decreased mast cell density in the airways are not clear. Interestingly, no changes at the parenchymal level were observed in any of the groups. These results could be explained by our cases being mostly exudative or early proliferative cases. Liebler et al. previously analysed ARDS cases in different histological stages, reporting that mast cells were increased only at a later fibroproliferative stage, and they suggested that these cells were not implicated in fibrogenesis [33].

IL-17 is known to be involved in the early pathogenesis of both viral and non-viral DAD, mainly due to its capacity to regulate neutrophil influx. Increased levels of IL-17 in the lungs are believed to be an early indicator of disease severity in H1N1 virus infection [34–36]. We found increased levels of IL-17 in both groups but surprisingly only at the small airway level, showing significant correlations with neutrophil and macrophage cell densities in ARDS group. Our finding reinforced the contribution of bronchioles to the pathogenesis of acute interstitial diseases by secreting cytokines, and inflammatory mediators involved in the early phases of acute lung injury [21, 22].

This study had several limitations. Due to its retrospective character, we had limited access to clinical and ventilatory data at the time of death, especially from the A(H1N1)pdm09 cases, because some of the patients presented with very severe disease and died within a few days in other institutions [12], and they were referred to our service for autopsy. We were not able to include a group of patients with non-viral and pulmonary ARDS, given the heterogeneity of this group (bacterial, fungi, lung cancer, immunosuppression, etc), that would make interpretation of data difficult. Therefore, we cannot conclude about the specificity of the immune response in H1N1 cases. Furthermore, we analysed lung tissue from patients who developed severe disease, which might have presented differently in less severe cases of DAD and could explain the lack of significant clinical-morphological correlations. In both DAD groups we had cases with secondary bacterial infection, which might have influenced the results. Nevertheless, we were able to identify a cytotoxic profile compatible with viral infection in H1N1 cases.

## Conclusions

In summary, we described the immune cellular profiles of DAD cases due to viral A(H1N1)pdm09 and non-viral DAD in fatal cases. Both DAD groups had increased numbers of neutrophils and macrophages, but the H1N1 group had a cellular cytotoxic profile at the parenchymal level, with a divergent pattern of dendritic and mast cell depletion at the small airway level. Other immune cell alterations were present only at the small airway level in non-viral DAD cases, reinforcing the role of small airways in the pathogenesis of exudative ARDS. Autopsy material is a unique material that, by providing access to different parts of the lungs, enhance our understanding of the immune processes occurring in DAD.

## Additional files

Additional file 1: **Table S1.** Antibodies and processing used in immunohistochemical analyses. **Table S2.** Cell densities of studied markers in the lung parenchyma of the Control, ARDS and H1N1 groups without significant difference. **Table S3.** Cell densities of studied markers in the small airways of the Control, ARDS and H1N1 groups without significant difference. (DOCX 17 kb)

Additional file 2 **Figure S1.** Representative zoomed photomicrographs showing positive stained cells for: CD8+ cells (A), CD4+ cells (B), CD57+ cells (C), Granzyme A (D), tryptase + cells (E), CD207+ cells (F), CD83+ cells (G), IL17+ cells (H), neutrophil elastase + cells (I), CD68+ cells (J). (TIFF 11074 kb)

## Abbreviations

ARDS: Acute respiratory distress syndrome
BM: Basement membrane
DAD: Diffuse alveolar damage
DCs: Dendritic cells
H&E: Haematoxylin and eosin
NK cells: Natural Killer cells
PaO_2_/FiO_2_: Ratio of arterial oxygen tension to the fraction of inspired oxygen
PEEP: Positive end-expiratory pressure
