# Supplementary material for: Airway and parenchyma immune cells in influenza A(H1N1)pdm09 viral and non-viral diffuse alveolar damage
Source: Respir Res. 2017 Aug 3;18:147. doi: 10.1186/s12931-017-0630-x (PMC5543730; doi:10.1186/s12931-017-0630-x)
Supplement: Supplementary file 1 — Table S1. Antibodies and processing used in immunohistochemical analyses. Table S2. Cell densities of studied markers in the lung parenchyma of the Control, ARDS and H1N1 groups without significant difference. Table S3. Cell densities of studied markers in the small airways of the Control, ARDS and H1N1 groups without significant difference. (DOCX 17 kb) [file 12931_2017_630_MOESM1_ESM.docx]

**Additional file 1**

**Table S1.**

| **Antibodies and processing used in immunohistochemical analyses.** | | | | |
| --- | --- | --- | --- | --- |
| **Antibody** | **Pre-treatment** | **Clone** | **Dilution** | **Origin** |
| **CD4** | Citrate | aB12 | 1:200 | Novocastra, Benton Lane/United Kingdom |
| **CD8** | Citrate | C8/144B | 1:400 | Dako, Glostrup/Denmark |
| **CD20** | Citrate | L 26 | 1:20000 | Dako, Glostrup/Denmark |
| **CD57** | Citrate | TB01 | 1:200 | Dako, Glostrup/Denmark |
| **Granzyme B** | Citrate | polyclonal | 1:4000 | Spring Bioscience, California/USA |
| **Granzyme A** | Citrate | GA-6 | 1:100 | Sanquim, Amsterdam/The Netherlands |
| **Chymase** | Citrate | cc1 | 1:5000 | Novocastra, Benton Lane/United Kingdom |
| **Tryptase** | Citrate | AA1 | 1:20000 | Dako, Glostrup/Denmark |
| **CD83** | Tris-Citrate | 1H4B | 1:100 | Vector Laboratories, California/USA |
| **CD207** | Citrate | 12D6 | 1:600 | Abcam, Cambridge/United Kingdom |
| **IL-17** | Citrate | polyclonal | 1:3000 | R&D, Minneapolis/USA |
| **Foxp3** | Citrate | 236A/E7 | 1:200 | Abcam, Cambridge/United Kingdom |
| **Neutrophil** | none | NP57 | 1:300 | Dako, Glostrup/Denmark |
| **CD68** | Citrate | KP1 | 1:1000 | Dako, Glostrup/Denmark |

**Table S2.**

| **Cell densities of studied markers in the lung parenchyma of the Control, ARDS and H1N1 groups**  **without significant difference.** | | | |  |
| --- | --- | --- | --- | --- |
| **Markers** (cells10^3^/µm) | **Control** | **ARDS** | **H1N1** |  |
| **Granzyme B** | **6 ± 4.1** | **7.9 ± 3.6** | **8.3 ± 4.7** |  |
| **CD20** | **0.7 [0.6]** | **0.5 [0.7]** | **0.7 [0.5]** |  |
| **Chymase mast cells** | **0.5 [0.8]** | **0.6 [0.6]** | **0.7 [1.1]** |  |
| **Tryptase mast cells** | **3 ± 1.5** | **2.2 ± 1.5** | **3.2 ± 1.7** |  |
| **IL-17** | **14.9 ± 6.6** | **16.9 ± 6.2** | **15.6 ± 7** |  |
| Data are expressed as median [IQRs] or mean ± SD. | | | | |

| **Table S3.**  **Cell densities of studied markers in the small airways of the Control, ARDS and H1N1 groups without significant difference.** | | | |  |
| --- | --- | --- | --- | --- |
| **Markers** (cells10^3^/µm) | **Control** | **ARDS** | **H1N1** |  |
| **CD4** | **17.5 ± 10.4** | **15.1 ± 9.8** | **22.4 ± 17** |  |
| **CD8** | **22.2 ± 13.2** | **35.7 ± 24.8** | **47 ± 33.4** |  |
| **CD20** | **8.1[16.6]** | **3.7 [9.6]** | **9.4 [10.2]** |  |
| **CD 57** | **5.6 [2.2]** | **2.9 [1.4]** | **3.5 [2.6]** |  |
| **Granzyme B** | **33.2 [16.1]** | **30.9 [22.5]** | **39.5 [38.3]** |  |
| **Granzyme A** | **8.7 [8.4]** | **7.6 [14.3]** | **32 [38.9]** |  |
| **Chymase mast cells** | **8.8 [7.5]** | **6.9 [5.3]** | **5.8 [6.9]** |  |
| Data are expressed as median [IQRs] or mean ± SD. | | | | |
