# Supplementary figures and images for: Airway and parenchyma immune cells in influenza A(H1N1)pdm09 viral and non-viral diffuse alveolar damage
Source: Respir Res. 2017 Aug 3;18:147. doi: 10.1186/s12931-017-0630-x (PMC5543730; doi:10.1186/s12931-017-0630-x)

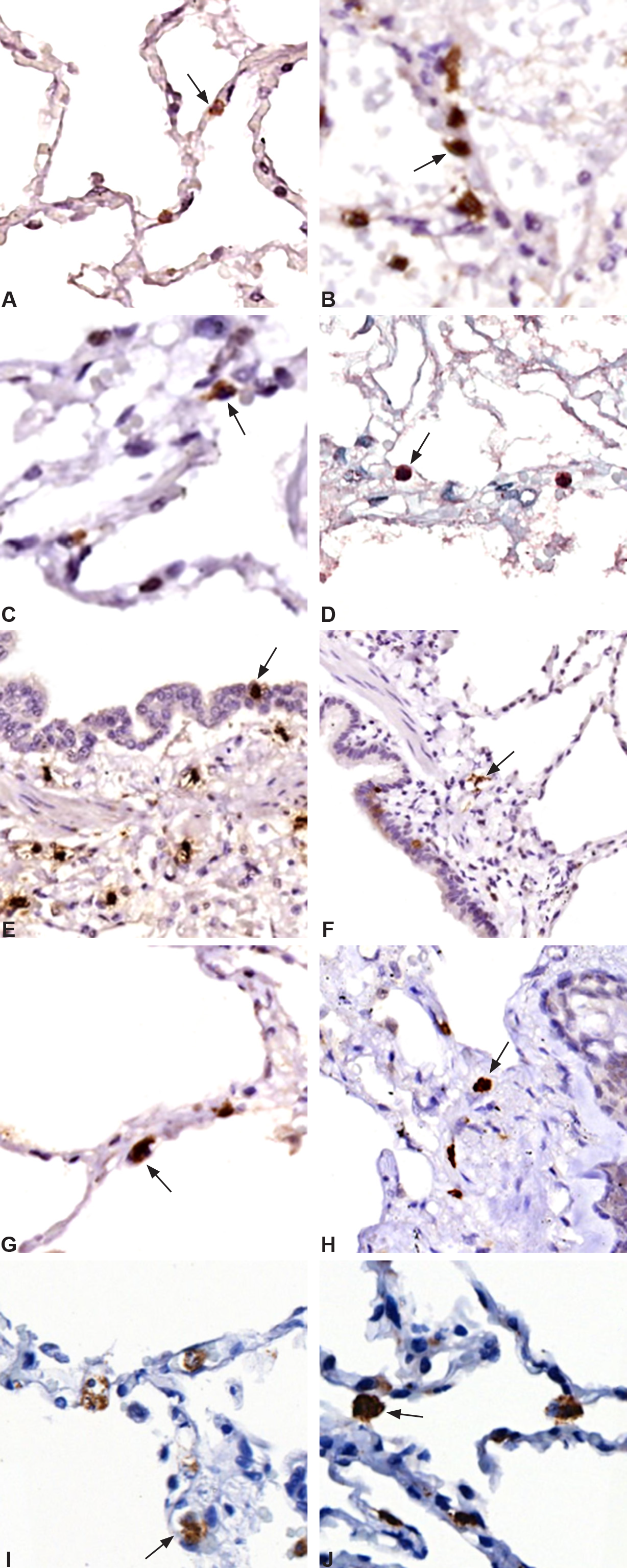

Supplement: Supplementary file 2 — Figure S1. Representative zoomed photomicrographs showing positive stained cells for: CD8+ cells (A), CD4+ cells (B), CD57+ cells (C), Granzyme A (D), tryptase + cells (E), CD207+ cells (F), CD83+ cells (G), IL17+ cells (H), neutrophil elastase + cells (I), CD68+ cells (J). (TIFF 11074 kb) [file 12931_2017_630_MOESM2_ESM.tif]
